# Evaluation of Phenolic Compounds and Pigments Content in Yellow Bell Pepper Wastes

**DOI:** 10.3390/antiox11030557

**Published:** 2022-03-15

**Authors:** María del Carmen Razola-Díaz, Ana Mª Gómez-Caravaca, Julia López de Andrés, Ana Voltes-Martínez, Alberto Zamora, Gema M. Pérez-Molina, David J. Castro, Juan Antonio Marchal, Vito Verardo

**Affiliations:** 1 Department of Nutrition and Food Science, University of Granada, Campus of Cartuja, 18071 Granada, Spain; carmenrazola@ugr.es; 2 Department of Analytical Chemistry, University of Granada, Campus of Fuentenueva, 18071 Granada, Spain; anagomez@ugr.es; 3 Biomedical Research Center, Institute of Nutrition and Food Technology ‘José Mataix’, University of Granada, Avda del Conocimiento sn., 18100 Armilla, Spain; 4 Centre for Biomedical Research (CIBM), Biopathology and Regenerative Medicine Institute (IBIMER), University of Granada, 18100 Granada, Spain; julialdeandres@correo.ugr.es (J.L.d.A.); anavolmar@correo.ugr.es (A.V.-M.); jmarchal@ugr.es (J.A.M.); 5 Instituto de Investigación Biosanitaria ibs.GRANADA, University Hospitals of Grana-da-University of Granada, 18100 Granada, Spain; 6 Excellence Research Unit “Modeling Nature” (MNat), University of Granada, 18100 Granada, Spain; 7 BioFab i3D—Biofabrication and 3D (Bio)Printing Laboratory, University of Granada, 18100 Granada, Spain; 8 Unidad de Lípidos y Riesgo Vascular, Servicio de Medicina Interna, Hospital de Blanes, Corporació de Salut del Maresme i la Selva, 17300 Blanes, Spain; azamora@salutms.cat; 9 Grupo de Medicina Traslacional y Ciencias de la Decisión, Departamento de Ciencias Médicas, Facultad de Medicina, Universidad de Girona, 17004 Girona, Spain; 10 Grupo Epidemiología Cardiovascular y Genética, CIBER, Enfermedades Cardiovasculares (CIBERCV), 08003 Barcelona, Spain; 11 Department I+D+i Vellsam Materias Bioactivas S.L., 04200 Tabernas, Spain; gperez@vellsam.com (G.M.P.-M.); dcastro@vellsam.com (D.J.C.)

**Keywords:** *Capsicum annuum* L., recovery of bioactive compounds, phenolic acids, flavonoids, HPLC-MS, antioxidant activity

## Abstract

Bell peppers are one of the most important species consumed and cultivated in Spain. Peppers are a source of carotenoids and phenolic compounds widely associated with biological activities such as antimicrobial, antiseptic, anticancer, counterirritant, cardioprotective, appetite stimulator, antioxidant, and immunomodulator. However, undersized and damaged fruits are usually wasted. Thus, in order to evaluate the phenolic content, a Box–Behnken design has been carried out to optimize the extraction from *Capsicum annuum* yellow pepper by ultrasound-assisted extraction (UAE). The independent factors were time (min), ethanol/water (% *v*/*v*) and solvent/sample ratio (*v*/*w*). The model was validated by ANOVA and confirmed. Furthermore, the whole pepper and the pepper without peduncles and seeds were extracted using optimal conditions and characterized by HPLC-ESI-TOF-MS. Moreover, their antioxidant activities, measured by three different methods (DPPH, ABTS, and FRAP), carotenoid composition, assessed by HPLC-MS, and chlorophyll content, assessed by a spectrophotometric method, were compared. A total of 38 polar compounds were found of which seven have been identified in pepper fruit extracts for the first time. According to the results, whole pepper (WP) samples presented higher content in phenolic acids; meanwhile, the edible portion (EP) was higher in flavonoids. No differences were found in the antioxidant activity except for the FRAP assay where the WP sample showed higher radical scavenging activity. EP samples showed the highest content of carotenoids and WP ones in chlorophylls.

## 1. Introduction

*Capsicum* peppers from the *Solanaceae* family are a diverse genus including more than 31 species from which five are domesticated: *Capsicum baccatum*, *Capsicum annuum*, *Capsicum pubescens*, *Capsicum frutescens*, and *Capsicum chinense*. Between them, *C. annuum* bell peppers are the most important species consumed and cultivated in Spain for their pungency and unique flavor. Further, from 2008 to 2019, the bell pepper consumption ranged from over 210 million kilograms to around 240 million kilograms in Spain. Furthermore, the community of Andalusia reached the highest amount of produced fresh peppers in Spain in 2019 with a total volume of more than 979.8 thousand metric tons of fresh peppers produced (Statista). Furthermore, it is a vegetable of great importance in nutrition because it is a source of pigments, vitamins, and phenolic compounds widely associated with biological activities such as antimicrobial, antiseptic, anticancer, counterirritant, cardioprotective, appetite stimulator, antioxidant, and immunomodulatory [1,2]. The extraction step is of significant importance for the isolation and separation of those phenolic compounds and depends on factors such as extraction time, temperature, and solvent. In *Capsicum* fruits, different traditional methods and solvents have been evaluated previously as maceration and Soxhlet methods; however, low recoveries were obtained [3]. Furthermore, recently, there has been an increment in the demand for extracting techniques that reduce extraction times and consumption of organic solvents. Therefore, among other extraction methods, ultrasound-assisted extraction is considered an excellent green alternative that has been widely used for the extraction of phenolic compounds in other matrices [4,5]. Some authors have reported using ultrasound technology in pepper matrices but with different approaches such as for extracting hydroxy-sanshool compounds [6], capsaicinoids [7,8,9,10,11,12], capsinoids [13], β-cyclodextrin [14], minerals (Ca, Fe, K, Mg, Mn, P, and Zn) [15], oils [16], oleoresin [17], or alkenylbenzenes [18], or with other uses such as for removing the residual solvent from fragrant oils [19] or for improving drying kinetics [20,21,22].

During the phase of selection of bell peppers, some of them are discharged because they are damaged or undersized. However, they contain bioactive compounds that could be used for animal feed or as nutraceutical, cosmeceutical, and functional food ingredients. Thus, the present study focused on developing and optimizing the ultrasound-assisted extraction of total phenolic compounds from bell pepper wastes and to compare the phenolic, antioxidant, carotenoid, and chlorophyll content in whole pepper (including peduncles and seeds) and in the edible portion.

## 2. Materials and Methods

### 2.1. Chemicals

Double-deionized water was from Millipore (Bedford, MA, USA). Gallic acid, Trolox (6-hydroxy-2,5,7,8-tetra-methylchromen-2-carboxylic acid), DPPH (2,2-diphenyl-1-picrylhydrazyl), ABTS (2,2′-azino-bis(3-ethylbenzothiazoline-6-sulfonic acid) diammonium salt) and FRAP (ferric reducing antioxidant power) reagents as TPTZ (2,4,6-Tri(2-pyridyl)-1,3,5-triazine); standards vanillic acid, chlorogenic acid, ferulic acid, quercetin, catechin, rutin, and carotenoid standards fucoxanthin, violaxanthin, neoxanthin, astaxanthin, antheraxanthin, meso-zeaxanthin, zeaxanthin, lutein, canthaxanthin, echinenone, and β-carotene were purchased from Sigma-Aldrich (St. Louis, MO, USA). Na_2_CO_3_ was purchased from BDH AnalaR (Poole, UK). HPLC-grade water, Folin-Ciocalteu reagent, and butylated hydroxytoluene were purchased from Merck KGaA (Darmstadt, Germany).

### 2.2. Sample Preparation

**Figure 1:**
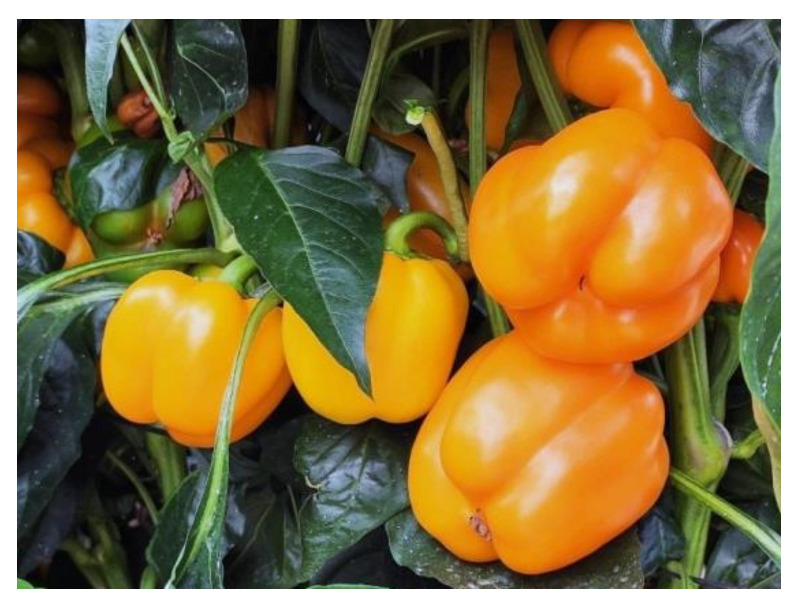
*Capsicum annuum* var. Kioto yellow pepper.

Samples were obtained from two companies located in Almería (Spain) that are clients of Vellsam. There were two samples of 3 kg of *Capsicum annuum* var. Kioto yellow pepper (Figure 1) which were coded as 1 and 2 corresponding to different companies.

The samples were lyophilized entire (WP) (whole pepper including edible portion, peduncles, and seeds) and only the edible portion (EP) After the lyophilization, the samples were milled and sieved with an average particle size of 0.2 mm. They have been named as given in Table 1.

### 2.3. Experimental Design

A Box-Behnken design was established to optimize the extraction of phenolic compounds with high antioxidant activity from pepper edible parts. It is composed of 15 experiments structured in three blocks with three levels (−1, 0, 1). Each experiment was carried out in duplicate. The independent variables were time (5, 45, and 85 min), ratio ethanol/water (0, 50, and 100% *v*/*v*), and ratio solvent/sample (10, 105, and 200 *v*/*w*) and the dependent variable considered the total phenolic content measured by the Folin-Ciocalteu method. It was adjusted to a second-order polynomial model equation and ANOVA was performed to evaluate the adjustment of the model. Response surface methodology (RSM) was used to select the optimal conditions. All data were processed using STATISTICA 7.0 (2002, StatSoft, Tulsa, OK, USA).

### 2.4. Ultrasound-Assisted Extraction

The extractions were performed by using an ultrasonic bath (Bandelin, Sonorex, RK52, Berlin, Germany) operating at a frequency of 35 kHz. Briefly, each extraction was carried out by adding ethanol/water solution (10 mL) with a determined amount of pepper and sonicated for a fixed time according to the established by the model. After the extraction, the samples were centrifuged at 3500 rpm for 15 min and the supernatant was stored at −18 °C until the analysis.

### 2.5. Determination of Total Phenolic Content (TPC)

TPC of pepper extracts was determined by the Folin-Ciocalteu spectrophotometric method [23]. Thus, 100 µL of the extract was added to 500 µL of the Folin-Ciocalteu reagents and 6 mL of bi-distilled water. The flask was agitated for a minute. After that, 2 mL of 15% (*w*/*v*) Na_2_CO_3_ was added and made up to 10 mL with bi-distilled water. The flasks were kept in darkness. After 2 h, measures at 750 nm and 25 °C were carried out with a UV-visible spectrophotometer (Spectrophotometer 300 Array, UV-Vis, single beam, Shimadzu, Duisburg, Germany). Gallic acid was used for the calibration curve from 1 to 500 ppm. Results are expressed as mg gallic acid equivalents (GAE)/g dry weight (d.w.).

### 2.6. Antioxidant Activity: DPPH, ABTS and FRAP

Three different assays were used to determine the antioxidant capacity of the pepper extracts obtained with the optimal conditions. In all assays, Trolox was used as the standard for the calibration curves and the results were expressed in mg of Trolox equivalents (TE)/g of dry weight (d.w.). For the DPPH assay, 0.1 mL of the samples was added to 2.9 mL of 100 μM DPPH methanol solution and measured after 30 min at 517 nm at 25 °C [24,25]. For the ABTS as described previously by Pellegrini et al. [26], the assay consisted of adding 10 μL of extracts to 1 mL of ethanol diluted ABTS reagent and measuring the decrease in absorbance after 30 min at 734 nm at 30 °C. Finally, the FRAP assay was performed according to a method previously described by Pulido et al. [27]. Briefly, the FRAP reagent was prepared by mixing 25 mL of 0.3 mM acetate buffer (pH 3.6); 2.5 mL of 10 mM of TPTZ in 40 mM HCl solution, and 2.5 mL of 20 mM FeCl_3_∙6H_2_O solution. The analyses were performed by adding 90 μL of water and 0.9 mL of FRAP reagent to 30 μL of the extracts and measuring the absorbance after incubation at 37 °C for 30 min at 595 nm.

### 2.7. Determination of Phenolic Compounds in Pepper Extracts by HPLC-ESI-TOF-MS Analysis

Polar compounds present in the pepper extracts, obtained with the optimized conditions, were analyzed using an Acquity Ultra Performance Liquid Chromatography (UPLC) system (Waters Corporation, Milford, MA, USA) coupled to an electrospray ionization (ESI) source operating in the negative mode and a mass detector time of flight (TOF) micro mass spectrometer (Waters). The compounds of interest were separated on an ACQUITY UPLC BEH Shield RP18 column (1.7 µm, 2.1 × 100 mm; Waters Corporation, Milford, MA, USA) at 40 °C using a gradient previously used by Verni et al. [28]. H_2_O acidified with 1% of acetic acid and acetonitrile were used as phase A and B, respectively.

MassLynx 4.1 software (Waters Corporation, Milford, MA, USA) was used for elaborating the data. An indicative base peak total ion chromatogram of the pepper samples analyzed by HPLC-MS is attached as Figure S1. To quantify the phenolic compounds identified in pepper samples, five calibration curves were made: vanillic acid, chlorogenic acid, ferulic acid, quercetin, and rutin. Table 2 contains the standards used, with their calibration ranges and curves, the regression coefficients, and the limits of detection (LOD) and quantification (LOQ). The calibration curves were elaborated by using the peak areas of each standard measured by HPLC at different concentrations. Calibration ranges were determined previously according to the LOQ values. The regression coefficients were >0.99 in all cases which means that all calibration curves had good linearity. LOD ranged between 0.17 and 2.76 μg/mL, and LOQ between 0.57 and 9.20 μg/mL.

### 2.8. Carotenoid Analysis by HPLC-MS

Carotenoids were extracted according to the protocol described by Castro-Puyana et al. 2013 [29] with some modifications. Briefly, 10 mg of lyophilized and powdered pepper was added to 1.5 mL of ethanol containing 0.1% (*v*/*w*) of butylated hydroxytoluene. The mixture was centrifuged for 10 min at 10,000 (4 °C). The extracts were collected and filtered through 0.2 μm nylon syringe filters and stored at −18 °C until the analysis. Pepper extracts were analyzed by an Acquity UPLC coupled to a XEVO-TQ-S triple quadrupole mass spectrometry (Waters Corporation, Milford, MA, USA). Carotenoids were separated on a YMC-C30 column (250 × 4.6 mm 3 µm). The mobile phases consisted of methanol with 5% water and 0.1% formic acid as mobile phase A and methyl tert-butyl ether as mobile phase B. The conditions of the solvent gradient were 60% A to 0% A in 30 min with a flow rate of 1 mL min^−1^. MRM transitions of each carotenoid were obtained by infusion of the analytical standards in positive ionization mode. Additional mass spectrometric parameters were as follows: source temperature: 150 °C, desolvation temperature: 500 °C, cone gas flow: 150 °C, source offset: 30 V, desolvation gas flow: 1000 L/h, collision gas flow: 0.15 mL min^−1^, and collision gas: argon. The data were acquired using MassLynx version 4.1 (Waters, San Jose, CA, USA). Carotenoids were quantified by standards of violaxanthin, neoxanthin, meso-zeaxanthin, zeaxanthin, lutein, lycopene, and β-carotene (Table 3). The calibration curves were prepared from the limit of quantification (LOQ) to 500–625 µg/L. All calibration curves revealed a good linearity among different concentrations, and the determination coefficients of the linear regression were higher than 0.99 in all cases. The method used for analysis showed a limit of detection (LOD) within the range 0.02–1.18 µg/L and the LOQ was within 0.08–3.96 µg/L.

### 2.9. Chlorophylls Analysis

The content of chlorophyll pigments a and b was determined following the procedure described by Porra et al. (1989) [30]. Briefly, the samples were extracted by acetone/water 80% in an ultrasound bath for 15 min. Next, the samples were centrifuged at 9000 rpm for 10 min. The supernatant was collected and filtered through 0.2 µm filters. Then, the absorbance of the extract samples, appropriately diluted, was measured at 647, 664, and 750 nm on a UV–Vis spectrophotometer (Perkin-Elmer Lambda 25). The results, expressed as µg/mL, were calculated using the equations:

$$\mathrm{Chlorophyll}\;a=12.25A^{664}-2.55A^{647}$$

$$\mathrm{Chlorophyll}\;b=20.31A^{647}-4.91A^{664}$$

$$\mathrm{Chlorophylls}\;\left(a+b\right)=17.76A^{647}+7.34A^{664}$$

where A is the absorbance at the established wavelengths. All absorbance measurements must have the absorbance at 750 nm (A^750^) subtracted.

### 2.10. Statistical Analysis

Statistical differences were calculated by Tukey’s test using STATISTICA software version 7.0 (2002, StatSoft, Tulsa, OK, USA).

## 3. Results and Discussion

### 3.1. Fitting the Model

A Box–Behnken design combined with RSM was used to establish the optimal conditions of time (X_1_), ethanol/water ratio (X_2_), and solvent/sample ratio (X_3_) in ultrasound bath technology to extract phenolic compounds from pepper edible parts. The experimental values of the total phenolic content (TPC) for each run are presented in Table 4. The observed results ranged from 2.5 to 11.6 mg GAE/g d.w., which correspond to the extraction conditions 5 min and 100% ethanol, and 45 min and 50% ethanol, respectively, both at a solvent/sample ratio of 105 *v*/*w*. In fact, all the runs using 100% ethanol as solvent reported lower recovery of TPC.

The experimental data were analyzed adjusting it to a second-order polynomial equation, a regression model that provides the lowest residual value using the least-squares method. All regression coefficients are shown in Table 5. With a significance level of *p* < 0.05, the lineal terms of time (β_1_) and ratio ethanol/water (β_2_), the crossed between them (β_12_), and all the quadratic terms (β_11_, β_22_, β_33_) had a significant effect. After discarding the non-significant terms, the model was recalculated and tested by ANOVA. As can be seen in Table 5, the model revealed a high correlation between the response variable and the factors (R^2^ = 0.9173), a good fit to the regression model (*p* < 0.05), and an insignificant lack of fit (*p* > 0.05); therefore, as reported by Bezerra et al. (2008) [31], the adequacy of the model is confirmed.

**Figure 2:**
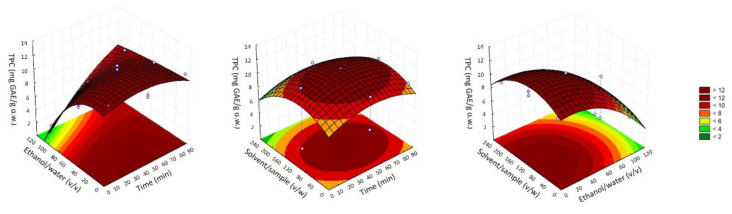
Response surface plots showing combined effects of process variables time vs. EtOH/H_2_O ratio (on the **left**), time vs. sample/solvent ratio (in the **middle**), and EtOH/H_2_O ratio vs. sample/water ratio (on the **right**).

The selection of the optimal conditions was carried out by using RSM among the three-dimensional graphs as shown in Figure 2. Thus, a compromise between the minimum possible values of each independent factor was made. As can be seen, when increasing the percentage of ethanol higher than 40% combined with the effect of time or solvent/sample ratio, lower amounts of phenolic compounds were achieved. Regarding the combined effect of time with ratio solvent/sample intermedium, values of those two parameters allowed the highest recovery of TPC. Therefore, these observations made it possible to establish the best conditions as: 30 min, 20% ethanol/water, and ratio solvent/sample 120 *v*/*w*, which provided a predicted value of 11.18 ± 2.03 mg GAE/g d.w for TPC. Furthermore, the model was verified and did not report significant differences between the observed (11.26 ± 1.71 mg GAE/g d.w.) and predicted value (*p* < 0.05).

Dos Anjos et al. (2021) [32] optimized the extraction of phenolic compounds by ultrasound bath from malagueta pepper (*Capsicum frutescens*). They found as optimal conditions 95% methanol, ratio solvent/sample (*v*/*w*) 200, and 15 min at 55 °C with a result in the same range of magnitude reported here. Similarly, Herrera-Pool et al. (2021) [33] reported methanol as the better solvent for extracting phenolic compounds from *Capsicum Chinese* pepper leaves compared to acetone and hexane. However, those solvents are hazardous for human health and could not be used for nutraceuticals. In contrast, Rybak et al. (2020) [34], using water as a solvent in a ratio solvent/sample (*v*/*w*) of 4 for a total of 90 min at 20 °C, reported a value of 25.5 mg/g d.w. by ultrasound technology in red bell pepper, which demonstrated that water is a food-grade safe solvent that allowed the recovery of phenolic compounds from pepper. In the same way, Bae et al. (2012) [35] reported the maximum flavonoid recovery from pepper when using ethanol instead of methanol or N-N-dimethylformamide. Additionally, Dias et al. (2017) [36] confirmed that ethanol allowed the extraction of more antioxidant compounds than methanol by ultrasound bath technology from dedo de moça pepper (*Capsicum baccatum* L.). Farahmandfar et al. (2017) [37] optimized the extraction of phenolic compounds from *Capsicum frutescens* pepper by comparing various techniques and reported that ultrasound bath technology at similar conditions as those reported in this study (ethanol 50%, for 20 min at 50 °C) allowed a higher phenolic recovery than supercritical CO_2_ and traditional solvent extraction methods. Compared to the results reported in *C. annuum* yellow-orange varieties, Carvalho et al. (2015) [38] analyzed eight *Capsicum* pepper genotypes including *C. annuum*, the yellow variety Pimenta Amarela, in which they reported a total phenolic content of 4.8 mg/g d.w. Hamed et al. (2019) [39], in *C. annuum* cultivar flavorburst, obtained a value of 5.4 mg/g d.w. Chávez-Mendoza et al. (2015) [40] reported a total phenols content from 0.7 to 1.11 mg/g d.w. in *C. annuum* var. Orangela/Terrano. In this context, the optimized ultrasound conditions allowed the achievement of a high recovery of phenols from pepper by using a food-grade and non-hazardous solvent, ethanol/water 20:80, avoiding thermal degradation as no temperature was used during the process, and which is less time consuming than other conventional methods.

### 3.2. HPLC Analysis of Polar Compounds in Pepper Extracts

A total of 38 polar compounds have been tentatively identified in the pepper optimal extracts. Between them, there are organic acids, amino acids, terpenoids, lignan derivates, phenolic acids, flavonoids, and other metabolites. Table 6 shows an overview of all proposed compounds with their retention time (min), molecular formula, experimental and calculated *m/z*, and *m/z* fragments. Furthermore, all metabolites showed a score higher than 90% and an error (ppm) lower than 5. These parameters were given by the software MassLynx 4.1. To identify the compounds, the generated molecular formulas and some in-source fragments were checked, studied, and compared with different databases such as PubChem, Mass bank, Phenol-Explorer, and the literature.

Three organic acids were identified. Peak 2, at 0.49 min and *m/z* 191, was recognized as quinic acid; peak 3, at 0.59 min and *m/z* 175, was identified as ascorbic acid; and peak 4, at 0.63 min and *m/z* 191, was identified as citric acid. At peak 5 and 7 with *m/z* 164 and 203, two amino acids were identified, DL-phenylalanine and tryptophan, respectively. Pantothenic acid (vitamin B5) was found at 1.21 min corresponding to peak 6. Furthermore, a lignan derivate was identified with an *m/z* of 521 (peak 29) named lariciresinol glucopyranoside. The results were in concordance with the previous literature [33,41,42].

Considering the phenolic acids that are the main representative group in pepper extracts, four hydroxybenzoic acids and five hydroxycinnamic acids were found. *p*-Hydroxybenzoic acid β-glucoside (peak 8), vanillic acid 1-O-β-D-glucopyranosyl ester (peak 9), homovanillic acid hexoside (peak 10), feruloyl-hexoside (peak 13), and sinapic acid-O-hexoside (peak 14) corresponding to *m/z* 299, 329, 343, 355, and 385, respectively, were identified. Additionally, peaks 11 and 22, with the molecular formula C_15_H_18_O_9_, were identified as caffeic acid 4-O-β-D-glucopyranoside or 1-O-caffeoyl-β-D-glucopyranoside isomers a and b, respectively. All data are in agreement with previous studies [33,41,42]. Two phenolic acids have been tentatively identified here for the first time. Firstly, peak 1, with the molecular formula C_13_H_12_0_11_ and *m/z* fragment 217, corresponding to the molecular formula C_7_H_6_O_8_ (tri-hydroxy gallic acid), was a galloyl ester tentatively identified as galloyl-1,4-galactarolactone previously detected, but not quantified in fruits [43]. Furthermore, the compound found at 9.11 min (peak 30) with the *m/z* fragments 191 and 353 was tentatively named 1,5-di-O-caffeoylquinic acid previously reported in artichoke [44] and spices [45].

Flavonoids are the more numerous group of phenolic compounds found in the pepper samples analyzed. Corresponding with peaks 16 and 20, two isomers (a and b) of quercetin dihexoside were identified. With an *m/z* of 579 at peaks 19 and 24, the compounds luteolin-6-C-β-D-glucopyranoside-8-C-α-L-arabinopyranoside or luteolin-7-O-[2-(β-D-apiofuranosyl)-β-D-glucopyranoside isomers a and b, respectively, were found. Two isomers of apigenin-7-O-β-D-apiofuranosyl (1 → 2)-β-D-glucopyranoside or apigenin-8-C-α-L-arabinoside 6-C-β-D-glucoside were detected at 7.41 and 8.29 min (peak 23 and 27). Corresponding with the molecular formula C_21_O_20_H_11_ and an *m/z* of 447, three isomers (a, b, and c) of quercetin-3-rhamnopyranoside or luteolin-8-glucoside were identified at peaks 25, 26, and 35. Additionally, compounds found at 4.97, 6.74, 7.20, 8.85, 9.38, 9.55, and 10.96 min (peaks 12, 17, 21, 28, 31, 32, and 36) were tentatively named quercetin-3-vicianoside, quercetin-3,7-di-O-α-L-rhamnopyranoside, quercetin-3-rutinoside-7-glucoside, phloretin dihexoside, diosmetin-7-O-β-D-glucoside, rutin pentoside, and luteolin-7-O-(2-apiofuranosyl-4-glucopyranosyl-6-malonyl) glucopyranoside, respectively. All previously described flavonoids are in agreement with the literature [33,41,42]. Moreover, three flavonoids have been reported in whole pepper or edible pepper here for the first time. The first compound, at peak 34, with an *m/z* of 405 and the molecular formula C_19_H_18_O_10_ is 5,7,2’,4’-tetrahydroxy-3,6,8,5’-tetramethoxyflavone according to its *m/z* fragment 285 that corresponds to 3’,4’,5,7-tetrahydroxyflavone. It was tentatively named erigeroflavanone according to Nam et al. (2008) who reported it for the first time in flowers of *Erigeron annuus* attributing it to have protein glycation and aldose reductase inhibitory activity [46]. At 11.15 min (peak 37), a kaempferol derivate with the kaempferol *m/z* fragment 285 was detected that, according to the PubChem database, was named kaempferol 3-(3’’-acetyl-alpha-L-arabinopyranosyl)-glucoside in concordance with other authors that reported similar compounds in pepper leaves [32]. Moreover, the compound with an *m/z* of 665 (peak 38) was luteolin-7-(2-O-apiosyl-6-O-malonyl) glucoside as reported previously in *Capsicum chinense* pepper leaves [33] and sweet red peppers [46].

**Table 6:** Polar compounds tentatively identified in pepper extracts by HPLC-ESI-TOF-MS.

No.	Tentative Identification	Rt (min)	Molecular Formula	*m*/*z* Experimental	*m*/*z* Calculated	*m/z* Fragments	Score	Error (ppm)	Source
1	Galloyl-1,4-galactarolactone	0.41	C_13_H_12_O_11_	343.0306	343.0301	217.0026	95.4	1.5	[43]
2	Quinic acid	0.49	C_7_H_12_O_6_	191.0553	191.0556	-	99.9	1.6	[33,41,42]
3	Ascorbic acid	0.59	C_6_H_8_O_6_	175.0236	175.0243	-	100.0	−4.0	[33,41,42]
4	Citric acid	0.63	C_6_H_8_O_7_	191.0190	191.0192	-	100.0	−1.0	[33,41,42]
5	DL-phenylalanine	0.92	C_9_H_10_NO_2_	164.0718	164.0717	-	98.4	0.6	[33,41,42]
6	Pantothenic acid	1.21	C_9_H_17_NO_5_	218.1027	218.1034	146.0818; 88.0379	97.6	−3.2	[33,41,42]
7	Tryptophan	1.47	C_11_H_11_N_2_O_2_	203.0763	203.0767	-	96.2	−2.0	[33,41,42]
8	p-Hydroxybenzoic acid β-glucoside	1.75	C_13_H_16_O_8_	299.0771	299.0772	137.0221	99.6	−0.3	[33,41,42]
9	Vanillic acid 1-O-β-D-glucopyranosylester	2.51	C_14_H_18_O_9_	329.0859	329.0873	167.0345	92.8	−4.3	[33,41,42]
10	Homovanillic acid hexoside	2.76	C_15_H_20_O_9_	343.1035	343.1029	166.0290	96.6	1.7	[33,41,42]
11	Caffeic acid 4-O-β-D-glucopyranoside or 1-O-Caffeoyl-β-D-glucopyranoside isomer a	3.69	C_15_H_18_O_9_	341.0818	341.0814	179.0349	93.2	1.2	[33,41,42]
12	Quercetin-3-vicianoside	4.97	C_26_H_30_O_16_	597.1435	597.1456	-	91.0	−3.5	[33,41,42]
13	Feruloyl hexoside	5.08	C_16_H_20_O_9_	355.1021	355.1029	175.0372	94.3	−2.3	[33,41,42]
14	Sinapic acid-O-hexoside	5.58	C_17_H_22_O_10_	385.1131	385.1135	205.0483	98.1	−1.0	[33,41,42]
15	Deoxyloganic acid isomer a	5.78	C_16_H_24_O_9_	359.1333	359.1342	153.0903; 197.0790	98.0	−2.5	[47], PUBCHEM
16	Quercetin dihexoside isomer a	6.22	C_27_H_30_O_16_	609.1453	609.1454	427.1250; 299.0568	97.3	−0.2	[33,41,42]
17	Quercetin-3,7-di-O-α-L-rhamnopyranoside	6.74	C_27_H_30_O_15_	593.1514	593.1506	387.1404; 363.0728; 353.0635; 427.1241	95.2	1.3	[33,41,42]
18	Deoxyloganic acid isomer b	6.79	C_16_H_24_O_9_	359.1327	359.1342	153.0908; 197.0781	91.9	−4.2	[47], PUBCHEM
19	Luteolin-6-C-β-D-glucopyranoside-8-C-α-L-arabinopyranoside or Luteolin-7-O-[2-(β-D-apiofuranosyl)-β-D-glucopyranoside isomer a	6.89	C_26_H_28_O_15_	579.1340	579.1350	459.0906; 399.0709; 369.0592	92.2	−1.7	[33,41,42]
20	Quercetin dihexoside isomer b	7.10	C_27_H_30_O_16_	609.1444	609.1456	463.0849; 299.0161	99.7	−2.0	[33,41,42]
21	Quercetin-3-rutinoside-7-glucoside	7.20	C_33_H_40_O_21_	771.1990	771.1984	609.1482; 341.0884; 301.0323	95.2	0.8	[33,41,42]
22	Caffeic acid 4-O-β-D-glucopyranoside or 1-O-Caffeoyl-β-D-glucopyranoside isomer b	7.28	C_15_H_18_O_9_	341.0878	341.0873	179.0321	91.4	1.5	[33,41,42]
23	Apigenin-7-O-β-D-apiofuranosyl-β-D-glucopyranoside or Apigenin-8-C-α-L-arabinoside 6-C-β-D-glucoside isomer a	7.41	C_26_H_28_O_14_	563.1397	563.1401	473.1078; 383.0772; 353.0643; 299.0193	98.5	−0.7	[33,41,42]
24	Luteolin-6-C-β-D-glucopyranoside-8-C-α-L-arabinopyranoside or Luteolin-7-O-[2-(β-D-apiofuranosyl)-β-D-glucopyranoside isomer b	7.50	C_26_H_28_O_15_	579.1334	579.1350	489.1024; 399.0685; 369.0590	93.3	−2.8	[33,41,42]
25	Quercetin-3-rhamnopyranoside or Luteolin-8-glucoside isomer a	7.64	C_21_H_20_O_11_	447.0907	447.0927	357.0572; 327.0481; 299.0513	92.1	−4.5	[33,41,42]
26	Quercetin-3-rhamnopyranoside or Luteolin-8-glucoside isomer b	7.95	C_21_H_20_O_11_	447.0919	447.0927	357.0609; 327.0482; 299.0550; 285.0394	99.7	−1.8	[33,41,42]
27	Apigenin-7-O-β-D-apiofuranosyl-β-D-glucopyranoside or Apigenin 8-C-α-L-arabinoside 6-C-β-D-glucoside isomer b	8.29	C_26_H_28_O_14_	563.1401	563.1401	473.1059; 443.0989 383.0761; 353.0647	95.5	0.0	[33,41,42]
28	Phloretin dihexoside	8.85	C_27_H_34_O_15_	597.1800	597.1819	387.1019; 357.0962; 327.0537	96.5	−3.2	[33,41,42]
29	Lariciresinol glucopyranoside	8.99	C_26_H_34_O_11_	521.2012	521.2023	-	97.4	−2.1	[33,41,42]
30	1,5-di-O-Caffeoylquinic acid	9.11	C_25_H_24_O_12_	515.1192	515.1190	353. 1074; 271.0197; 191.0330	96.9	0.4	[44,45]
31	Diosmetin-7-O-β-D-glucoside	9.38	C_22_H_22_O_11_	461.1094	461.1084	298.0460	94.4	2.2	[33,41,42]
32	Rutin pentoside	9.55	C_32_H_38_O_20_	741.1860	741.1878	579.1342; 285.0375	93.2	−2.4	[33,41,42]
33	Menthol glucuronide	9.63	C_16_H_28_O_7_	331.1755	331.1757	155.1442; 137.0984	99.6	−0.6	[47]
34	Erigeroflavanone	10.29	C_19_H_18_O_10_	405.0820	405.0822	285.0396; 217.0034	96.8	−0.5	[46]
35	Quercetin-3-rhamnopyranoside or Luteolin-8-glucoside isomer c	10.74	C_21_H_20_O_11_	447.0914	447.0927	300.0250; 271.0227; 255.0271	99.8	−2.9	[33,41,42]
36	Luteolin-7-O-(2-apiofuranosyl-4-glucopyranosyl-6-malonyl) glucopyranoside	10.96	C_35_H_40_O_23_	827.1899	827.1882	783.1999; 285.0384	99.9	2.1	[33,41,42]
37	Kaempferol-3-(3’’-acetyl-alpha-L-arabinopyranosyl)-glucoside	11.15	C_28_H_30_O_16_	621.1478	621.1456	411.1345; 285.0383	99.8	3.5	[32] PUBCHEM
38	Luteolin-7-(2-O-apiosyl-6-O-malonyl)-glucoside	11.17	C_29_H_30_O_18_	665.1367	665.1354	285.0376	97.2	2.0	[33,46]

Finally, two terpenoids have been found and reported here for the first time. Corresponding with peaks 15 and 18, two isomers of a monoterpene iridoid named deoxyloganic acid were detected according to the *m/z* fragment 197 according to the MassBank record: PR309193 by Tsugawa et al. (2019) [48] and the PubChem database. Similarly, the compound found at 9.63 min (peak 33) with the *m/z* fragment 155 corresponding to menthol was named menthol glucuronide, previously described as a tea metabolite [47].

### 3.3. Quantification of Phenolic Compounds in Pepper Extracts by HPLC-MS

A total of 9 phenolic acids and 19 flavonoids were quantified in WP and EP extracts obtained with the optimized conditions (Table 7). The analyses were made in triplicate.

As can be seen in Table 7, WP samples had a higher content of total phenolic compounds than EP samples; however, some differences between them should be highlighted. In general, phenolic acids were the most representative polyphenols accounting for 66–93% of the total phenolic content. WP showed a higher content of total phenolic acids with feruloyl glucose being the main compound followed by sinapic acid-O-hexoside and vanillic acid 1-O-β-D-glucopyranosylester. In the EP, the major phenolic acid was sinapic acid-O-hexoside followed by galloyl-1,4-galactarolactone and feruloyl glucose. Caffeic acid derivatives also had a significant impact on the phenolic acid content in both types of samples. No significant differences were found between samples 1 and 2 in WP nor in EP. In contrast, EP samples showed a higher content of total flavonoids than WP. The major flavonoid in all cases was kaempferol-3-(3’’-acetyl-alpha-L-arabinopyranosyl)-glucoside. Moreover, high amounts of quercetin, luteolin, and apigenin derivatives were found. Furthermore, some differences were found between samples 1 and 2. Samples coded as 2 had around half of the total flavonoid content of 1. This is mainly attributed to the slight differences in the environmental conditions due to the different locations of the culture that can affect the stress or non-stress conditions of the plant and consequently promote the production of different metabolites. Chel-guerrero et al. (2021) [49] compared two habanero peppers grown in black and red soils of Yucatán, México, and obtained values of total phenolic compounds ranging from 0.56 to 1.54 mg/g d.w. Dos Anjos et al. (2021) [32], in *C. frutescens* peppers from Brazil, reported a phenolic content from 15 to 17 mg/g d.w. and total flavonoids content from 6.1 to 7.8 mg/g d.w. Guclu et al. (2021) [43] found a total phenolic content of 368.77–496.07 µg/g in sweet red pepper samples. The obtained results are in the same range of magnitude as those previously reported by other authors in other pepper samples, and it can be confirmed that the origin and variety significantly affected the content in phenolic compounds.

### 3.4. Antioxidant Activity of Pepper Extracts by DPPH, ABTS and FRAP Assays

The antioxidant activity of the extracts obtained with the ultrasound optimal conditions was measured by three different methods and the results are summarized in Table 8. The values ranged from 11.58–12.44, 9.66–11.88, and 14.96–19.55 mg TE/g d.w. for the DPPH, ABTS, and FRAP, respectively. As reported by Ou et al. (2002) [50], polyphenols are chain-breaking antioxidants. Pepper extracts are rich in phenolic acids and flavonoids that are responsible for the antioxidant activity reported here.

No significant differences were found between EP and WP samples for DPPH and ABTS assays, but there were for FRAP. In this case, WP presented higher radical scavenging, reducing ferric anion. However, this lack of differences between the pepper without peel and the whole pepper may be due to its composition. EP has been found to be richer in flavonoids; meanwhile, WP was richer in the major phenolic component, phenolic acids. Therefore, these differences seem to compensate and give the sample the same antioxidant activity for the DPPH and ABTS assays. However, in contrast, for the FRAP assay, phenolic acids seemed to have the strongest influence. Compared with other authors, the results are in the range reported in other bell pepper varieties [3,40,51,52].

### 3.5. Carotenoid Composition of Pepper by HPLC-MS

The carotenoid content was analyzed in triplicate in each sample, and the obtained results are presented in Table 9. Carotenes are responsible for the yellow, red, and orange colors of fruits and vegetables, and offer beneficial health effects against various diseases such as cardiovascular disease, cancer, and other chronic diseases [40]. According to the results reported by de Azevedo-Meleiro and Rodriguez-Amaya [53], the main carotenoids in the studied pepper were violaxanthin, lutein, and β-carotene. Other minor compounds were zeaxanthin, α-carotene, neoxanthin, lycopene, and meso-zeaxanthin.

As seen in Table 9, EP samples showed higher amounts of carotenoids compared with the respective WP samples confirming that the localization of these compounds is in the edible portion of the fruit. Total carotenoid content ranged from 82.8 to 103.6 µg/g d.w. in EP samples and from 63.2 to 78.2 µg/g d.w. in WP samples. These data are in the same order of magnitude as those reported in the literature for yellow peppers [53], but lower than others reported by other authors [54,55].

It should be taken into account that, as reported by Jarén-Galán et al. (1999) [56], up to 30% of the pigments could be destroyed after post-harvesting due to autoxidation and the presence of pepper lipoxygenase enzyme. Moreover, the carotenoid content depends strongly on the variety and pedo-climatic conditions. However, several authors have reported results similar to those obtained here for *C. annuum* var. Kioto in other cultivars. Pugliese et al. (2013) [57] determined several carotenoids in 20 different genotypes from *C. annuum*, *C. baccatum*, *C. chacoense*, and *C. chinense* species. They reported ranges of 0.0–65.9 µg/g d.w. for violaxanthin, 0.0–2.3 µg/g d.w. for neoxanthin, 0.0–58.3 µg/g d.w. for antheraxanthin, 0.0–7.8 µg/g d.w. for lutein, 0.0–28.3 µg/g d.w. for zeaxanthin, and 0.0–43.5 µg/g d.w. for beta-carotene, and between them, zeaxanthin and lutein reported greater bio-accessibility. Howard et al. (2000) [58], in *C. annuum* cultivars, reported values of 0.0–21.27 µg/g d.w. in alpha-carotene and 3.4–8.0 µg/g d.w. in beta-carotene. Chávez-Mendoza et al. (2015) [40] reported a β-carotene content of 86.2 µg/g d.w. and lycopene content of 0.03 µg/g d.w. in *C. annuum* var. Orangela/Terrano. Carvalho et al. (2015) [38] reported values of 1.95–3.12, 0.08–4.6, 0.0–5.16, and 0.0–44.42 µg/g d.w. of lutein, zeaxanthin, alpha-carotene, and beta-carotene, respectively, in other varieties of *C. annuum*.

### 3.6. Chlorophylls Content of Pepper

The chlorophyll content was evaluated and the results are presented in Table 10. Total chlorophyll content ranged from 5.40 to 6.63 µg/g d.w. for the EP samples and from 25.13 to 27.79 µg/g d.w for the WPs. 

As can be seen from the results, yellow bell peppers showed a low chlorophyll content, not being their major pigment; however, differences can be appreciated among the samples. Those composed of the whole pepper had higher chlorophyll a and b content and total content in both cases. Besides there is a difference in the chlorophyll a/chlorophyll b ratio. WP samples had a ratio < 1 and EP samples had a > 1 ratio which indicates that chlorophyll a is mainly in the edible portion and chlorophyll b is present in the other parts of the peppers. Overall, these differences can be mainly attributed to the presence of peduncles and seeds in the whole pepper samples. There are few previous references of chlorophylls in yellow bell pepper samples. Ignat et al. (2013) [59] evaluated the effect of the maturity stage in the total chlorophyll content in intact yellow bell peppers and reported values in the same range as those obtained here for the mature ones, which additionally confirms the maturity stage of the analyzed peppers.

## 4. Conclusions

The ultrasound-assisted extraction of phenolic compounds from *C. annuum* var Kioto has been optimized using a Box–Behnken design combined with the RSM methodology. Optimal established conditions were 30 min, 20% ethanol, and a solvent/sample ratio of 120 *v*/*w*. The optimal extracts were characterized and a total of 38 polar compounds were identified from which seven were identified in pepper fruit extracts for the first time. Moreover, EP and WP were compared according to their phenolic compounds, antioxidant activity, and pigment content. WP samples presented a higher phenolic acids content; meanwhile, EP samples had a higher flavonoids content. No differences were found in the antioxidant activity except for the FRAP assay where the WP sample showed higher radical scavenging activity. Regarding pigments, it was noticed that carotenoids are much more concentrated in the edible portion of pepper, whereas chlorophylls are in the whole pepper.

Overall, the bell peppers that are usually wasted during the selection process of fruits could be considered a valuable raw material to obtain bioactive compounds such as phenolic compounds and carotenoids that can be used as functional food, nutraceutical, and cosmeceutical ingredients.

## Figures and Tables

**Table 1:** Samples and coded names.

Sample	Coded Name
Whole pepper sample 1	WP1
Edible Pepper sample 1	EP1
Whole pepper sample 2	WP2
Edible Pepper sample 2	EP2

**Table 2:** Standard analytes used for elaborating the calibration curves with the range used, equations, R^2^, LOD, and LOQ of each compound for the phenolic compounds analysis.

Standard	LOD (µg/mL)	LOQ (µg/mL)	Calibration Ranges (µg/mL)	Calibration Curves (µg/mL)	R^2^
Vanillic acid	0.3295	1.0985	LOQ-237	y = 30.182x − 74.233	0.9999
Chlorogenic acid	0.4572	1.5239	LOQ-247	y = 21.757x + 177.2	0.9913
Ferulic acid	1.1246	3.7486	LOQ-227	y = 8.8445x + 185.52	0.9924
Catechin	2.7612	9.2040	LOQ-230	y = 3.6022x + 112.4	0.9992
Rutin	0.2046	0.6821	LOQ-220	y = 48.608x + 138.63	0.9918
Quercetin	0.1739	0.5798	LOQ-227	y = 57.181x + 190.35	0.9904

**Table 3:** Standard analytes used for elaborating the calibration curves with the range used, equations, R^2^, LOD, and LOQ of each compound for the carotenoid analysis.

Standard	LOD (µg/L)	LOQ (µg/L)	Calibration Ranges (µg/L)	Calibration Curves (µg/L)	R^2^
Violaxanthin	1.1887	3.9622	LOQ-625	y = 100.941x + 574.666	0.9984
Neoxanthin	0.3817	1.2723	LOQ-625	y = 314.358x + 1231.63	0.9964
Meso-zeaxanthin	0.0247	0.0823	LOQ-500	y = 4861.13x + 4495.87	0.9958
Zeaxanthin	0.0270	0.0900	LOQ-625	y = 4443.93x + 5109.34	0.9970
Lutein	0.1244	0.4148	LOQ-500	y = 964.214x + 658.069	0.9958
β-Carotene	0.0228	0.0761	LOQ-535	y = 5254.84x + 2749.83	0.9976
Lycopene	0.3950	1.3166	LOQ-550	y = 303.78x − 243.955	0.9968

**Table 4:** Box–Behnken design with natural and coded values (parenthesis) of the conditions of extraction and the experimental results obtained for total phenolic content (TPC) expressed with the average and the standard deviation.

Run	Independent Factors			Response
	X^1^	X^2^	X^3^	TPC (mg GAE/g d.w.)
1	5 (−1)	0 (−1)	105 (0)	11.30 ± 0.04
2	85 (1)	0 (−1)	105 (0)	10.69 ± 0.22
3	5 (−1)	100 (1)	105 (0)	2.45 ± 0.15
4	85 (1)	100 (1)	105 (0)	8.83 ± 0.12
5	5 (−1)	50 (0)	10 (−1)	8.30 ± 0.09
6	85 (1)	50 (0)	10 (−1)	9.63 ± 0.19
7	5 (−1)	50 (0)	200 (1)	8.15 ± 0.08
8	85 (1)	50 (0)	200 (1)	7.47 ± 0.16
9	45 (0)	0 (−1)	10 (−1)	10.22 ± 0.05
10	45 (0)	100 (1)	10 (−1)	5.44 ± 0.07
11	45 (0)	0 (−1)	200 (1)	9.91 ± 0.03
12	45 (0)	100 (1)	200 (1)	6.41 ± 0.03
13	45 (0)	50 (0)	105 (0)	11.11 ± 0.02
14	45 (0)	50 (0)	105 (0)	11.03 ± 0.04
15	45 (0)	50 (0)	105 (0)	11.59 ± 0.05

X_1__–__3_: time (min), ethanol/water ratio (% *v*/*v*), and solvent/sample ratio (*v*/*w*).

**Table 5:** Estimated regression effects of the fitted second-order polynomial equation and ANOVA of the fitted model.

Regression Coefficients	Response			
	Effect	Standard Error	t-Value	*p*-Value
β_0_ *	8.235	0.0881	93.5063	0.0001
Lineal				
β_1_ *	2.034	0.2274	8.9440	0.0123
β_2_ *	−4.749	0.2157	−22.0161	0.0021
β_3_	−0.658	0.2274	−2.8949	0.1015
Crossed				
β_12_ *	3.497	0.3051	11.4625	0.0075
β_13_	−1.008	0.3051	−3.3058	0.0806
β_23_	0.637	0.3051	2.0875	0.1721
Quadratic				
β_11_ *	1.266	0.1588	7.9727	0.0154
β_22_ *	1.659	0.1588	10.4483	0.0090
β_33_ *	1.586	0.1588	9.9906	0.0099
R^2^	0.9173			
*p* model	0.0002			
*p* lack of fit	0.1184			

*: Significant at *p* < 0.05.

**Table 7:** Quantification of phenolic compounds in pepper samples by HPLC-MS (results are expressed as µg/g d.w.).

	WP1	EP1	WP2	EP2
**Total Phenolic Compounds**	3724.65 ± 13.97 ^a^	3241.45 ± 15.72 ^b^	3797.43 ± 12.87 ^a^	2766.99 ± 11.97 ^c^
**Total phenolic acids**	3188.05 ± 9.01 ^b^	2129.98 ± 6.80 ^c^	3526.68 ± 9.67 ^a^	2167.02 ± 6.55 ^c^
Galloyl-1,4-galactarolactone	399.55 ± 1.57 ^a^	366.97 ± 1.44 ^c^	399.27 ± 1.56 ^a^	383.17 ± 1.49 ^b^
*p*-Hydroxybenzoic acid ß-glucoside	133.80 ± 0.50 ^b^	172.22 ± 0.65 ^a^	133.52 ± 0.49 ^b^	105.93 ± 0.38 ^c^
Vanillic acid 1-O-ß-D-glucopyranosylester	465.48 ± 1.83 ^b^	226.80 ± 0.87 ^c^	586.99 ± 2.32 ^a^	222.77 ± 0.85 ^c, d^
Homovanillic acid hexoside	84.16 ± 0.30 ^b^	51.43 ± 0.16 ^a^	108.28 ± 0.39 ^b^	44.95 ± 0.14 ^a^
Caffeic acid 4-O-ß-D-glucopyranoside or 1-O-Caffeoyl-ß-D-glucopyranoside isomer a	260.60 ± 0.84 ^a^	140.19 ± 0.50 ^b^	258.84 ± 0.83 ^a^	118.77 ± 0.43 ^c^
Caffeic acid 4-O-ß-D-glucopyranoside or 1-O-Caffeoyl-ß-D-glucopyranoside isomer b	115.80 ± 0.43 ^a^	122.40 ± 0.45 ^c^	105.87 ± 0.40 ^b^	68.44 ± 0.29 ^d^
Feruloyl-hexoside	888.54 ± 0.98 ^b^	290.98 ± 0.39 ^d^	1131.00 ± 1.22 ^a^	428.13 ± 0.52 ^c^
Sinapic acid-O-hexoside	528.29 ± 1.59 ^c^	617.30 ± 1.84 ^a,b^	469.96 ± 1.42 ^d^	624.19 ± 1.85 ^a^
1,5-di-O-Caffeoylquinic acid	311.83 ± 0.98 ^b^	141.71 ± 0.50 ^d^	332.93 ± 1.04 ^a^	170.66 ± 0.58 ^c^
**Total flavonoids**	536.60 ± 4.96 ^c^	1111.47 ± 8.92 ^a^	270.75 ± 3.20 ^d^	599.97 ± 5.42 ^b^
Quercetin-3-vicianoside	<LOQ	2.41 ± 0.10	<LOQ	<LOQ
Quercetin dihexoside isomer a	16.60 ± 0.19 ^b^	28.25 ± 0.27 ^a^	<LOQ	16.67 ± 0.19 ^b^
Quercetin dihexoside isomer b	44.74 ± 0.38 ^c^	175.48 ± 1.26 ^a^	7.93 ± 0.13 ^d^	50.11 ± 0.41 ^b^
Quercetin-3,7-di-O-a-L-rhamnopyranoside	7.25 ± 0.13 ^b^	20.67 ± 0.22 ^a^	0.26 ± 0.08 ^d^	4.62 ± 0.11 ^c^
Luteolin-6-C-β-D-glucopyranoside-8-C-α-L-arabinopyranoside or Luteolin-7-O-[2-(β-D-apiofuranosyl)-β-D-glucopyranoside isomer a	41.18 ± 0.36 ^b^	58.42 ± 0.47 ^a^	10.10 ± 0.15 ^c^	40.17 ± 0.35 ^b^
Luteolin-6-C-β-D-glucopyranoside-8-C-α-L-arabinopyranoside or Luteolin-7-O-[2-(β-D-apiofuranosyl)-β-D-glucopyranoside isomer b	20.80 ± 0.22 ^b^	32.07 ± 0.29 ^a^	<LOQ	20.08 ± 0.21 ^b^
Quercetin-3-rutinoside-7-glucoside	<LOQ	23.92 ± 0.24	<LOQ	<LOQ
Apigenin-7-O-β-D-apiofuranosyl-β-D-glucopyranoside or Apigenin-8-C-α-L-arabinoside-6-C-β-D-glucoside isomer a	13.87 ± 0.17 ^b^	33.61 ± 0.31 ^a^	6.30 ± 0.12 ^d^	10.32 ± 0.15 ^c^
Apigenin-7-O-β-D-apiofuranosyl-β-D-glucopyranoside or Apigenin-8-C-α-L-arabinoside-6-C-β-D-glucoside isomer b	7.90 ± 0.13 ^b^	21.67 ± 0.23 ^a^	1.93 ± 0.09 ^d^	5.31 ± 0.12 ^c^
Quercetin-3-rhamnopyranoside or Luteolin-8-glucoside isomer a	13.95 ± 0.17 ^c^	24.77 ± 0.25 ^a^	0.63 ± 0.08 ^d^	16.78 ± 0.19 ^b^
Quercetin-3-rhamnopyranoside or Luteolin-8-glucoside isomer b	49.85 ± 0.41 ^c^	62.37 ± 0.50 ^a^	13.18 ± 0.17 ^d^	51.87 ± 0.43 ^b^
Quercetin-3-rhamnopyranoside or Luteolin-8-glucoside isomer c	13.84 ± 0.17 ^b^	172.85 ± 1.24 ^a^	<LOQ	5.87 ± 0.12 ^c^
Phloretin dihexoside	<LOQ	<LOQ	<LOQ	<LOQ
Diosmetin-7-O-β-D-glucoside	<LOQ	<LOQ	<LOQ	<LOQ
Rutin pentoside	52.24 ± 0.43 ^b^	51.15 ± 0.42 ^b^	20.10 ± 0.21 ^c^	65.65 ± 0.52 ^a^
Erigeroflavanone	47.67 ± 0.40 ^b^	43.53 ± 0.37 ^c^	55.25 ± 0.45 ^a^	28.92 ± 0.27 ^d^
Luteolin-7-O-(2-apiofuranosyl-4-glucopyranosyl-6-malonyl)-glucopyranoside	68.05 ± 0.54 ^c^	75.05 ± 0.58 ^b^	35.34 ± 0.32 ^d^	88.42 ± 0.67 ^a^
Kaempferol-3-(3’’-acetyl-alpha-L-arabinopyranosyl)-glucoside	105.43 ± 0.79 ^c^	214.62 ± 1.52 ^a^	87.95 ± 0.67 ^d^	148.19 ± 1.07 ^b^
Luteolin-7-(2-O-apiosyl-6-O-malonyl)-glucoside	33.22 ± 0.30 ^c^	70.62 ± 0.55 ^a^	31.77 ± 0.29 ^c^	46.99 ± 0.39 ^b^

Different letters (a–d) in the same line indicate significant differences (*p* < 0.05).

**Table 8:** Antioxidant activity of pepper extracts by DPPH, ABTS, and FRAP (results expressed as mg TE/g d.w. with the average ± standard deviation).

	WP1	EP1	WP2	EP2
DPPH	11.58 ± 0.13 ^a^	12.44 ± 0.02 ^a^	12.41 ± 0.39 ^a^	11.62 ± 0.00 ^a^
ABTS	9.66 ± 0.36 ^a^	11.88 ± 0.29 ^a^	11.28 ± 0.62 ^a^	10.45 ± 0.16 ^a^
FRAP	18.56 ± 0.08 ^b^	16.96 ± 0.81 ^a^	19.55 ± 0.54 ^b^	14.96 ± 0.15 ^a^

Different letters (a,b) in the same line indicate significant differences (*p* < 0.05).

**Table 9:** Carotenoid content in pepper measured by HPLC-MS/MS (results are expressed as µg/g d.w.).

	WP1	EP1	WP2	EP2
Violaxanthin	40.36 ± 0.14 ^b^	50.87 ± 0.10 ^a^	26.52 ± 0.07 ^d^	32.17 ± 0.09 ^c^
Neoxanthin	1.57 ± 0.02 ^b^	2.10 ± 0.04 ^a^	1.10 ± 0.00 ^d^	1.45 ± 0.00 ^c^
Meso-zeaxanthin	0.02 ± 0.00 ^c^	0.08 ± 0.00 ^b^	0.07 ± 0.00 ^b^	0.16 ± 0.00 ^a^
Zeaxanthin	6.50 ± 0.04 ^d^	9.00 ± 0.07 ^c^	15.40 ± 0.12 ^b^	20.10 ± 0.04 ^a^
Lycopene	0.61 ± 0.01 ^b^	0.82 ± 0.00 ^a^	0.12 ± 0.00 ^d^	0.26 ± 0.00 ^c^
Lutein	18.70 ± 0.09 ^b^	26.00 ± 0.06 ^a^	8.80 ± 0.03 ^d^	12.00 ± 0.06 ^c^
Alpha-carotene	2.45 ± 0.03 ^c^	3.04 ± 0.01 ^b^	3.09 ± 0.02 ^b^	4.06 ± 0.02 ^a^
Beta-carotene	7.96 ± 0.02 ^c^	11.73 ± 0.09 ^b^	8.08 ± 0.04 ^c^	12.65 ± 0.7 ^a^
Total carotenoids	78.17 ± 0.35 ^c^	103.61 ± 0.37 ^a^	63.18 ± 0.28 ^d^	82.85 ± 0.28 ^b^

Different letters (a–d) in the same line indicate significant differences (*p* < 0.05).

**Table 10:** Chlorophyl content in pepper (results are expressed as µg/g d.w.).

	WP1	EP1	WP2	EP2
Chlorophyll a	12.97 ± 0.12 ^d^	6.63 ± 0.09 ^b^	8.37 ± 0.03 ^c^	4.88 ± 0.13 ^a^
Chlorophyll b	14.81 ± 0.34 ^b^	1.38 ± 0.15 ^a^	16.75 ± 0.22 ^c^	0.52 ± 0.02 ^a^
Total chlorophylls	27.79 ± 0.46 ^d^	8.02 ± 0.24 ^b^	25.13 ± 0.25 ^c^	5.40 ± 0.15 ^a^

Different letters (a–d) in the same line indicate significant differences (*p* < 0.05).

## Data Availability

Data is contained within the article and Supplementary Materials.

## Supplementary Materials

The following supporting information can be downloaded at: https://www.mdpi.com/article/10.3390/antiox11030557/s1, Figure S1: Indicative base peak total ion chromatogram of the pepper samples analysed by HPLC-MS.
