# Supplementary figures and images for: Evaluation of Phenolic Compounds and Pigments Content in Yellow Bell Pepper Wastes
Source: Antioxidants (Basel). 2022 Mar 15;11(3):557. doi: 10.3390/antiox11030557 (PMC8944693; doi:10.3390/antiox11030557)

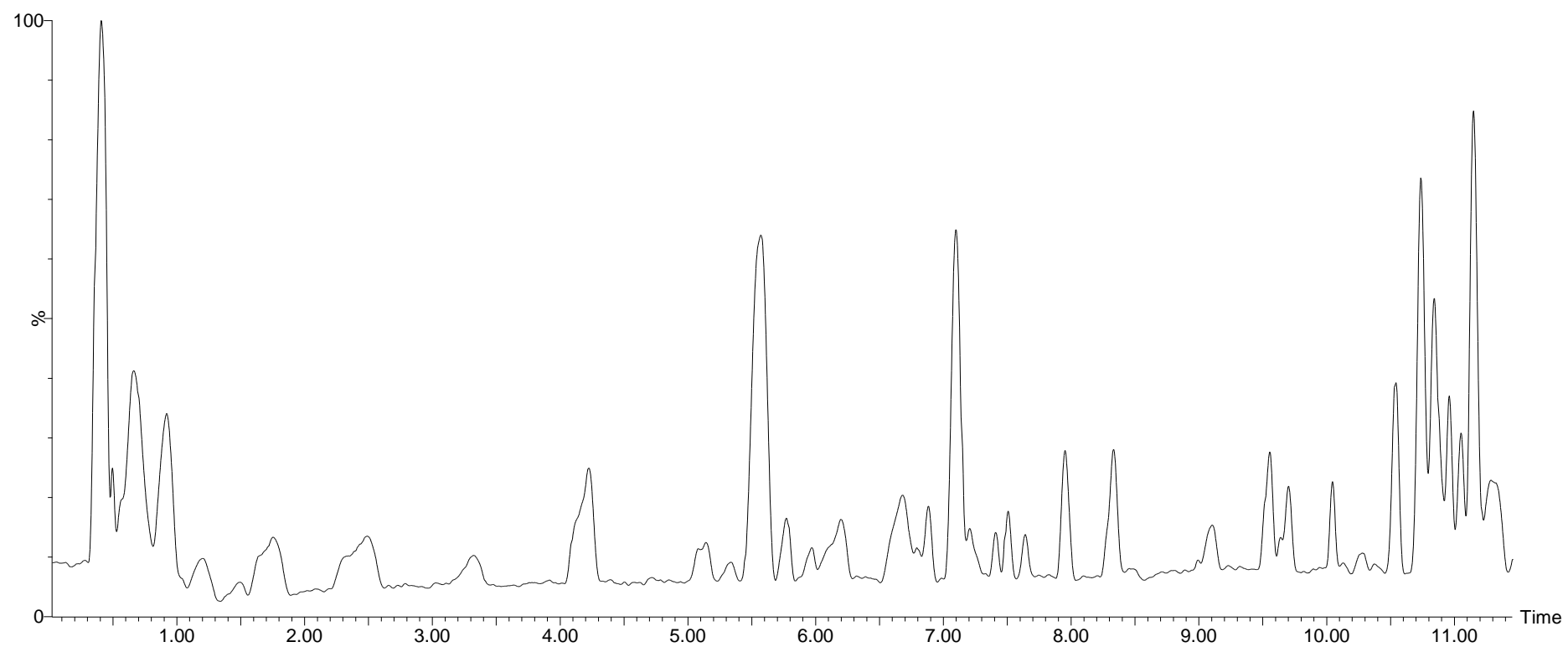

**Figure S1.** Indicative base peak total ion chromatogram of the pepper samples analysed by HPLC-MS.

Supplement: Supplementary file 1 [file antioxidants-11-00557-s001.zip › antioxidants-1620555-supplementary.pdf]
